# Deoxyshikonin Inhibits Viability and Glycolysis by Suppressing the Akt/mTOR Pathway in Acute Myeloid Leukemia Cells

**DOI:** 10.3389/fonc.2020.01253

**Published:** 2020-08-07

**Authors:** Huijuan Wu, Hongmian Zhao, Li Chen

**Affiliations:** ^1^Telemedicine and Connected Health Center, Huaihe Hospital of Henan University, Kaifeng, China; ^2^Department of Hematology, Huaihe Hospital of Henan University, Kaifeng, China

**Keywords:** deoxyshikonin, glycolysis, PKM2, the Akt/mTOR signaling, acute myeloid leukemia

## Abstract

Deoxyshikonin was reported to exhibit an anti-tumor effect in colorectal cancer. However, no studies are available to illustrate the effect of deoxyshikonin on acute myeloid leukemia (AML). The effects of deoxyshikonin on viability, apoptosis, caspase-3/7 activity, and cytochrome (Cyt) C expression were evaluated by Cell Counting Kit-8 assay, flow cytometry analysis, caspase-3/7 activity assay, and western blot analysis, respectively. Glucose consumption and lactate production were measured to determine the glycolysis level. The expression of pyruvate kinase M2 (PKM2) was detected by quantitative real-time polymerase chain reaction and western blot analysis. The results showed that deoxyshikonin inhibited cell viability and increased the apoptotic rate, the caspase-3/7 activity, and the Cyt C protein level in AML cells in a dose-dependent manner. Additionally, deoxyshikonin concentration-dependently decreased glucose consumption, lactate production, and PKM2 expression in AML cells. Deoxyshikonin inactivated the protein kinase B (Akt)/mammalian target of the rapamycin (mTOR) pathway. The activation of the Akt/mTOR pathway reversed the effects of deoxyshikonin on viability, apoptosis, and glycolysis in AML cells. In conclusion, deoxyshikonin dampened the viability and the glycolysis of AML cells by suppressing PKM2 *via* inactivation of the Akt/mTOR signaling.

## Introduction

Acute myeloid leukemia (AML), the most prevalent form of acute leukemia in adults, is an aggressive malignancy derived from hemopoietic progenitor cells and with poor survival rate and frequent relapse, posing a threat to the health and life of affected patients (1). AML is characterized by rapid growth, impaired apoptosis, and abnormal clonal accumulation of hematopoietic stem cells in the bone marrow due to various genetic and epigenetic changes, eventually leading to bone marrow failure (2). There were an estimated 19,520 new cases diagnosed with AML and 10,670 mortalities due to AML according to the statistics in 2018 (3). Presently, the efficacy of the standard chemotherapy for AML patients remains suboptimal, owing to drug resistance and high clinical relapse rate (4). In this regard, searching for novel anti-leukemia drugs is urgently required to effectively improve the outcome of patients with AML.

**Figure 1:**
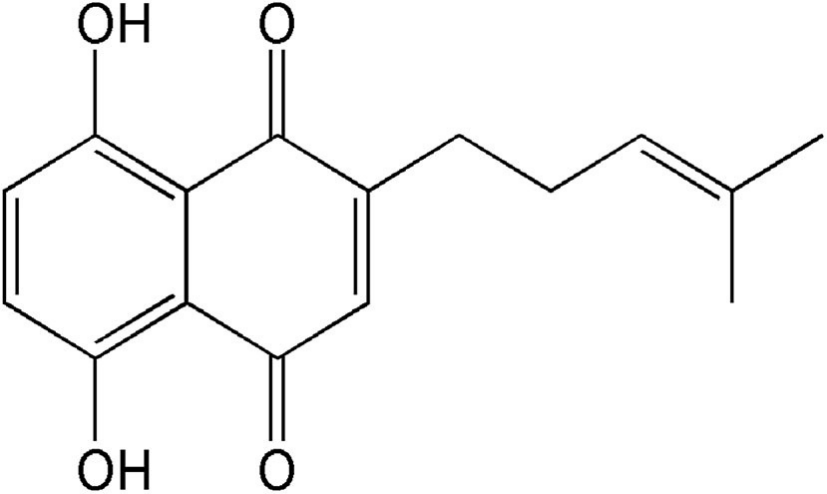
Chemical structure of deoxyshikonin.

Shikonin (5,8-dihydroxy-2-[(1S)-1-hydroxy-4-methylpent-3-en-1-yl]naphthalene-1,4- dione), a naturally occurring naphthoquinone extracted from the oriental traditional medical herb *Lithospermum erythrorhizon* Sieb. et Zucc., has been extensively used for the treatment of many diseases including burns, sore throats, HIV-1 infection, and macular eruption (5, 6). Currently, clinical and pharmacological properties studies have demonstrated that shikonin and its derivatives exhibit various biological activities, such as immune regulation and anti-thrombotic, anti-inflammatory, anti-oxidative, and anti-glycolytic activities (7, 8). An increasing number of researches reveal that shikonin derivatives have garnered much research interest due to its limited toxicity and stronger anti-tumor activities in miscellaneous cancers (9). Interestingly, a previous investigation proved that deoxyshikonin (its chemical structure is shown in Figure 1), a derivative of shikonin, exhibited an anti-tumor effect in colorectal cancer (10). However, no studies are available to illustrate the effect of deoxyshikonin on AML.

Aerobic glycolysis, also known as the Warburg effect, is well-recognized as a metabolic pathway in the rapidly proliferating cancer cells for the regeneration of energy and the biosynthesis of macromolecules even in the presence of sufficient oxygen (11). Aerobic glycolysis has been extensively accepted as an important characteristic of tumor cells including AML, eventually resulting in increased glucose consumption and lactate production (12, 13). Pyruvate kinase (PK) is a rate-limiting glycolytic enzyme. Pyruvate kinase M1 (PKM1) is expressed in normal differentiated tissues, whereas pyruvate kinase M2 (PKM2) is expressed in cancer cells, leading to increased glycolysis (14). As we all know, the protein kinase B (Akt)/mammalian target of rapamycin (mTOR) pathway widely existing in cells is one of the most important survival signaling pathways that participate in the regulation of diverse physiological processes, such as cell growth, apoptosis, and metabolism (15). It has been reported that the Akt/mTOR pathway is a positive regulator of PKM2 expression (16, 17). Previous studies suggested that shikonin inhibited glycolysis by suppression of PKM2 expression (8, 18, 19). Therefore, we hypothesized that deoxyshikonin inhibited viability and glycolysis, suppressing pyruvate kinase M2 *via* the Akt/mTOR pathway in acute myeloid leukemia cells.

In the present study, we assessed the effects and the underlying mechanisms of deoxyshikonin on viability, apoptosis, glycolysis, and PKM2 expression in AML cells. These results revealed that deoxyshikonin treatment inhibited viability, induced apoptosis, and suppressed glycolysis in AML cells. Mechanistically, the anti-AML effect of deoxyshikonin was mediated *via* repressing PKM2 by inactivation of the Akt/mTOR pathway.

## Materials and Methods

### Cell Cultivation and Treatment

AML cell lines, including THP-1 and HL60, were purchased from American Tissue Culture Collection (ATCC, Manassas, VA, USA) and routinely cultured in Roswell Park Memorial Institute-1640 medium (HyClone, South Logan, UT, USA) conjugated with 10% heat-inactivated fetal bovine serum (ExCell Bio, Shanghai, China) and 1% penicillin/streptomycin (Sigma-Aldrich, St Louis. MO, USA). The cells were fostered at 37°C in a drippy environment flushed with 95% air plus 5% CO_2_. Deoxyshikonin (purity >98%; Tauto Biotech, Shanghai, China) was dissolved in dimethyl sulfoxide (Sigma-Aldrich, St. Louis, MO, USA) as a reserve solution and diluted into different concentrations (0, 2.5, 5, 10, 20, and 40 μg/ml). The THP-1 and HL60 cells were exposed to diverse doses of deoxyshikonin for 48 h. The Akt-overexpressing plasmid (pcDNA-Akt) and the empty vector (pcDNA control) were obtained from Ribobio (Guangzhou, China). Transfection was performed using Lipofectamine 2000 reagent (Invitrogen, Carlsbad, CA, USA).

### Cell Counting Kit-8 Assay

Cell Counting Kit-8 (CCK-8) (Beyotime, Shanghai, China) was taken to evaluate the viability of AML cells. THP-1 and HL60 cells were seeded into 96-well plates at 2 × 10^3^ cells/well and dealt with various concentrations of deoxyshikonin (0, 2.5, 5, 10, 20, and 40 μg/ml) for 48 h or incubated with 20 μg/ml deoxyshikonin in the presence or the absence of 15 μM 740Y-P (Tocris Bioscience, Shanghai, China), an activator of the Akt/mTOR signaling pathway. After treatment for 48 h, 10 μl of CCK-8 solution was added to each well, followed by incubation for another 2 h. The optical density of each well was recorded at a wavelength of 450 nm using a microplate reader (Thermo Fisher Scientific, Waltham, MA, USA).

### Flow Cytometry Analysis for Apoptosis

After the treatments as aforementioned, annexin V FITC and propidium iodide kit (KeyGen, Nanjing, China) was implemented to analyze the apoptosis of THP-1 and HL60 cells. Cell apoptotic rate was detected by means of a flow cytometer (FACScan, BD Biosciences, San Diego, CA, USA).

### Caspase-3/7 Activity Assay

Following the treatments as aforementioned, Apo-ONE Homogeneous Caspase-Glo 3/7 Assay kit (Promega, Madison, WI, USA) was adopted to measure the caspase-3/7 activity of THP-1 and HL60 cells, referring to the manufacturer's description. Finally, M2000 Infinite Pro instrument (Tecan Trading AG, Maennedorf, Switzerland) was used to determine the luminescence.

### Determination of Glucose Consumption and Lactate Production

After the treatments as aforementioned, the culture medium of THP-1 and HL60 cells was collected for the measurement of glucose and lactate levels using a glucose uptake colorimetric assay kit (Sigma-Aldrich) and a lactic acid assay kit (Jiancheng Bioengineering Institute, Nanjing, China), respectively.

### PKM2 Activity Assay

PKM2 activity was detected by the lactate dehydrogenase-coupled assay as described earlier (8). Five microliters of whole-cell lysate was utilized in the assay. The absorbance at a wavelength of 340 nm was measured using a microplate reader (Thermo Fisher Scientific).

### Western Blot Analysis

The treated THP-1 and HL60 cells were collected and rinsed with phosphate-buffered saline, followed by the addition of ice-cold radioimmunoprecipitation assay lysis buffer (Beyotime) containing 1 mM phenylmethylsulfonyl fluoride (Sigma-Aldrich). A total of 20 μg of protein samples was fractionated on 10% sodium dodecyl sulfate-polyacrylamide gel electrophoresis prior to electro-transfer onto polyvinylidene difluoride membranes (Millipore, Bedford, MA, USA). After blocking with 5% defatted milk-Tris-based saline with Tween at room temperature for 2 h, the membranes were immunoblotted overnight at 4°C with corresponding primary antibodies against cytochrome C (Cyt C; Cell Signaling Technology, Inc., Danvers, MA, USA), phosphorylated Akt (p-Akt Ser473; Abcam, Cambridge, MA, USA), Akt (Abcam), glycogen synthase kinase-3β (GSK-3β; Abcam), phosphorylated GSK-3β (p-GSK-3β Ser9; Abcam), mTOR (Abcam), phosphorylated mTOR (p-mTOR Ser2448; Abcam), p70 ribosomal S6 kinase (p70S6K; Cell Signaling Technology, Inc.), phosphorylated p70S6K (p-p70S6K Thr389; Cell Signaling Technology, Inc.), eukaryotic translation initiation factor 4E-binding protein 1 (4EBP1; Cell Signaling Technology, Inc.), phosphorylated 4EBP1 (p-4EBP1 Thr70; Cell Signaling Technology, Inc.), PKM2 (Abcam), and β-actin (Abcam) and then incubated with horseradish peroxidase-conjugated secondary antibodies (Cell Signaling Technology, Inc.) for 1 h at room temperature. Lastly, an enhanced chemiluminescence kit (Amersham Pharmacia, Piscataway, NJ, USA) was implemented to examine the antigen–antibody complexes. β-Actin was used as a loading control. The protein bands were visualized by VersaDoc imaging system (Bio-Rad, Hercules, CA, USA).

### Quantitative Real-Time PCR

RNAiso Plus (TaKaRa, Dalian, China) was used to extract total RNA from treated THP-1 and HL60 cells, and the concentration of extracted RNA was measured using a NanoDrop 2000 spectrophotometer (Thermo Fisher Scientific). Reverse transcription was carried out using the PrimeScript RT Reagent Kit (Takara, Dalian, China). SYBR Green Taq Mix (TaKaRa) was then implemented to detect PKM2 mRNA expression on the StepOnePlus qPCR system (Thermo Fisher Scientific), with β-actin as an endogenous control. The thermocycling conditions were displayed as follows: 95°C for 30 s, followed by 35 cycles of 95°C for 5 s, 60°C for 30 s, and 70°C for 10 s. The primers were as follows: PKM2, 5′-GCTG CCAT CTAC CACT TGC-3′ (forward) and 5′-CCAG ACTT GGTG AGGA CGAT T-3′ (reverse); GAPDH, 5′-ATGT CGTG GAGT CTAC TGGC-3′ (forward); and 5′-TGAC CTTG CCCA CAGC CTTG-3′ (reverse). The 2^−ΔΔCt^ method was taken to quantify the expression level of PKM2 mRNA.

### Statistics

All data are shown as mean ± standard deviation of three independent experiments. Statistical assays were determined using SPSS 19.0 software (IBM Corp, Armonk, NY, USA), with Student's *t*-test or one-way ANOVA followed by Dunnett's test. *P* < 0.05 were regarded as statistically significant.

## Results

### Deoxyshikonin Inhibited the Viability of AML Cells

**Figure 2:**
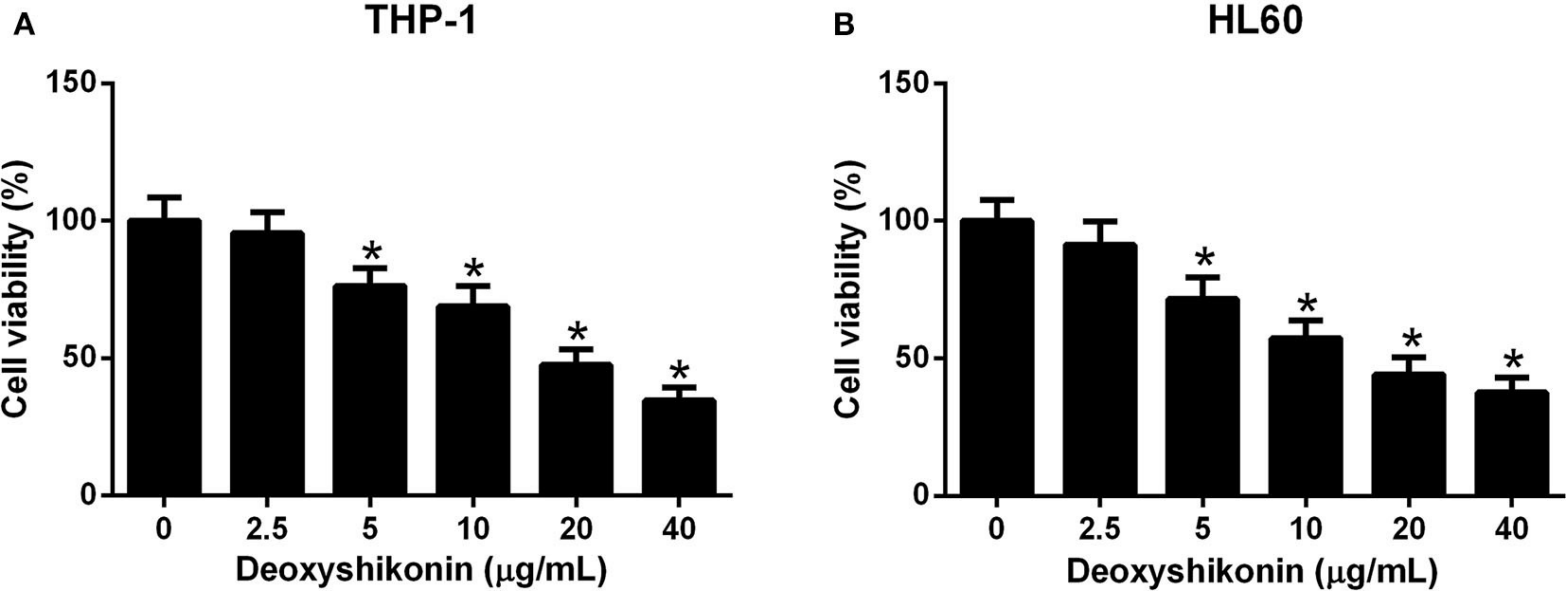
Deoxyshikonin dose-dependently inhibited the viability of acute myeloid leukemia cells. THP-1 **(A)** and HL60 **(B)** cells were administrated with various doses of deoxyshikonin (0, 2.5, 5, 10, 20, and 40 μg/ml) for 48 h, and cell viability was then assessed by CCK-8 assay. *P < 0.05 compared to the control group.

To clarify the anti-tumor activity of deoxyshikonin in AML cells, CCK-8 was taken to evaluate cell viability after THP-1 and HL60 cells were exposed to a series of deoxyshikonin concentrations (0, 2.5, 5, 10, 20, and 40 μg/ml) for 48 h. As shown in Figures 2A,B, cell viability was significantly declined in a concentration-dependent manner in THP-1 and HL60 cells in response to deoxyshikonin.

### Deoxyshikonin Enhanced the Apoptosis of AML Cells

**Figure 3:**
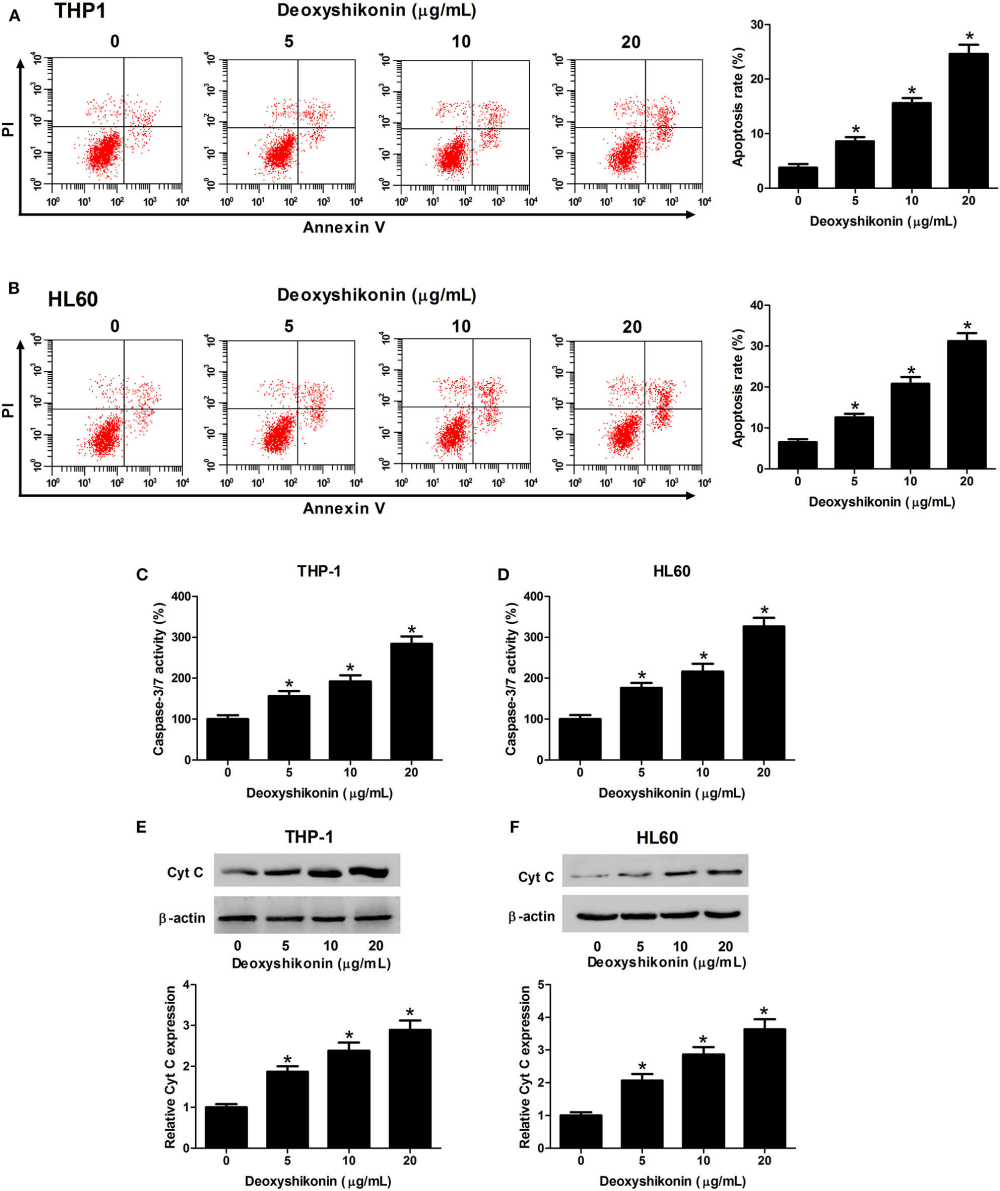
Deoxyshikonin concentration-dependently promoted the apoptosis of acute myeloid leukemia cells. THP-1 and HL60 cells were treated with increasing doses of deoxyshikonin (0, 5, 10, and 20 μg/ml), followed by detection of the apoptotic rate, caspase-3/7 activity, and Cyt C protein level by annexin V-FITC apoptosis assay **(A,B)**, caspase-3/7 activity assay **(C,D)**, and western blot analysis **(E,F)**, respectively. *P < 0.05 compared to the control group.

The effect of deoxyshikonin on the apoptotic consequences of AML cells was investigated by annexin V-FITC apoptosis assay. As a result, deoxyshikonin treatment led to a concentration-dependent increase of apoptotic rate in THP-1 (Figure 3A) and HL60 cells (Figure 3B). In line with the results of the flow cytometry analysis, an elevation of caspase-3/7 activity in deoxyshikonin-treated THP-1 (Figure 3C) and HL60 cells (Figure 3D) was observed. The mitochondrial protein Cyt C is known as the initiating factor of mitochondrial apoptosis pathway. The expression of apoptotic marker Cyt C in THP-1 and HL60 cells was further detected by western blot analysis. The results demonstrated that deoxyshikonin concentration-dependently increased Cyt C protein level in THP-1 (Figure 3E) and HL60 cells (Figure 3F) relative to the control group. These results suggested that deoxyshikonin facilitated the apoptosis of AML cells.

### Deoxyshikonin Suppressed Glycolysis in AML Cells

**Figure 4:**
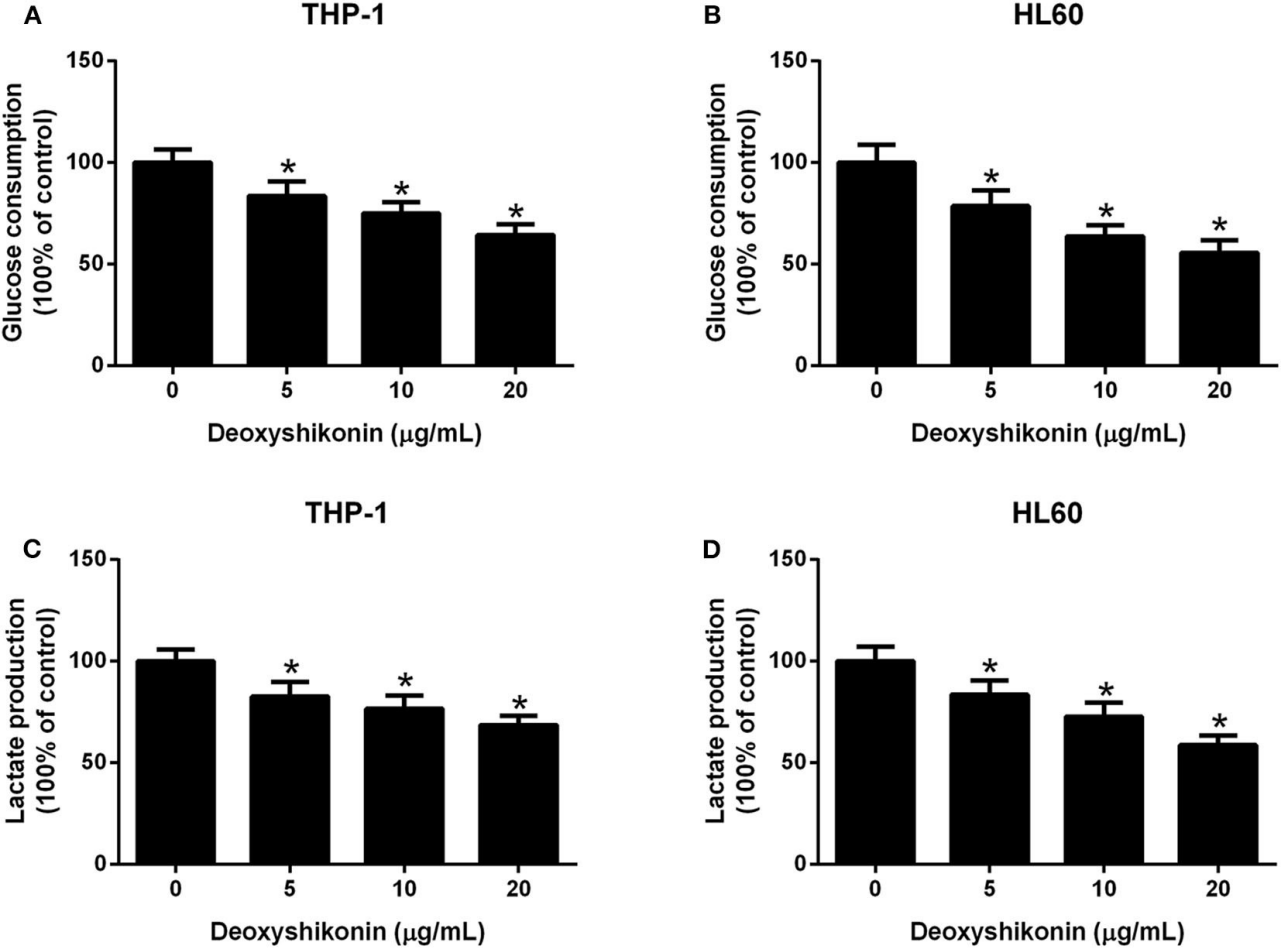
Deoxyshikonin concentration-dependently suppressed glycolysis in acute myeloid leukemia cells. After THP-1 and HL60 cells were administrated with increasing doses of deoxyshikonin (0, 5, 10, and 20 μg/ml) for 48 h, glucose consumption **(A,B)** and lactate production **(C,D)** were then measured. *P < 0.05 compared to control the group.

To further characterize the effect of deoxyshikonin on glycolysis in AML cells, we measured glucose consumption and lactate production in AML cells. These results demonstrated that deoxyshikonin exposure decreased glucose consumption (Figures 4A,B) and lactate production (Figures 4C,D) in THP-1 and HL60 cells in a dose-dependent manner. We concluded that deoxyshikonin suppressed glycolysis in AML cells.

### Deoxyshikonin Decreased the Expression Level of PKM2 in AML Cells

**Figure 5:**
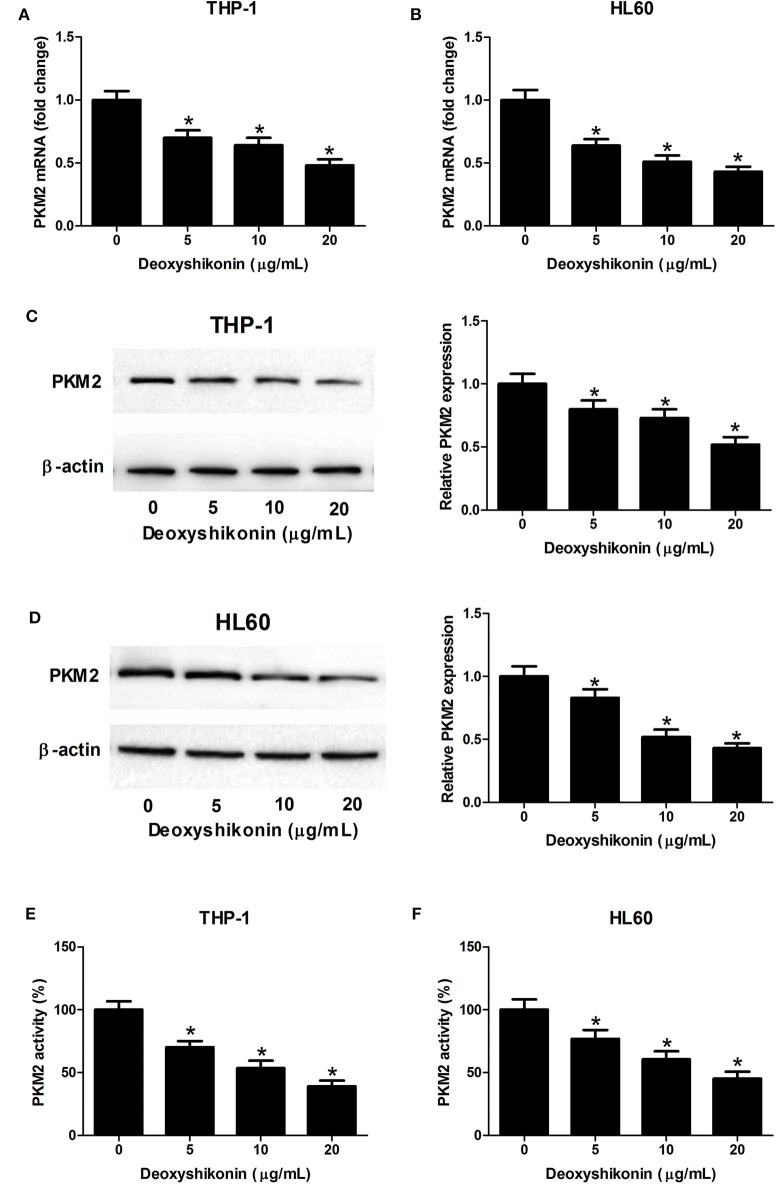
Deoxyshikonin decreased the expression level and the activity of PKM2 in acute myeloid leukemia cells. THP-1 and HL60 cells were exposed to increasing doses of deoxyshikonin (0, 5, 10, and 20 μg/ml) for 48 h. **(A–D)** The mRNA and protein levels of PKM2 were determined by qRT-PCR and western blot, respectively. **(E,F)** PKM2 activity was detected by the lactate dehydrogenase-coupled assay. *P < 0.05 compared to the control group.

A previous study reported that shikonin suppressed tumor aerobic glycolysis through suppressing the activity of PKM2 (18), an important rate-limiting enzyme in regulating cellular glycolysis (20). Accordingly, we supposed whether deoxyshikonin had an inhibitory effect on PKM2 expression. As expected, the quantitative real-time polymerase chain reaction (qRT-PCR) results showed that the mRNA levels of PKM2 were suppressed following the addition of deoxyshikonin in a dose-dependent manner in THP-1 (Figure 5A) and HL60 cells (Figure 5B). The western blot results showed that deoxyshikonin treatment inhibited the protein levels of PKM2 in a dose-dependent manner in THP-1 (Figure 5C) and HL60 cells (Figure 5D). We also found that deoxyshikonin suppressed PKM2 activity in THP-1 (Figure 5E) and HL60 cells (Figure 5F). These results suggested that deoxyshikonin decreased the expression level of PKM2 in AML cells.

### Deoxyshikonin Inactivated the Akt/mTOR Pathway in AML Cells

**Figure 6:**
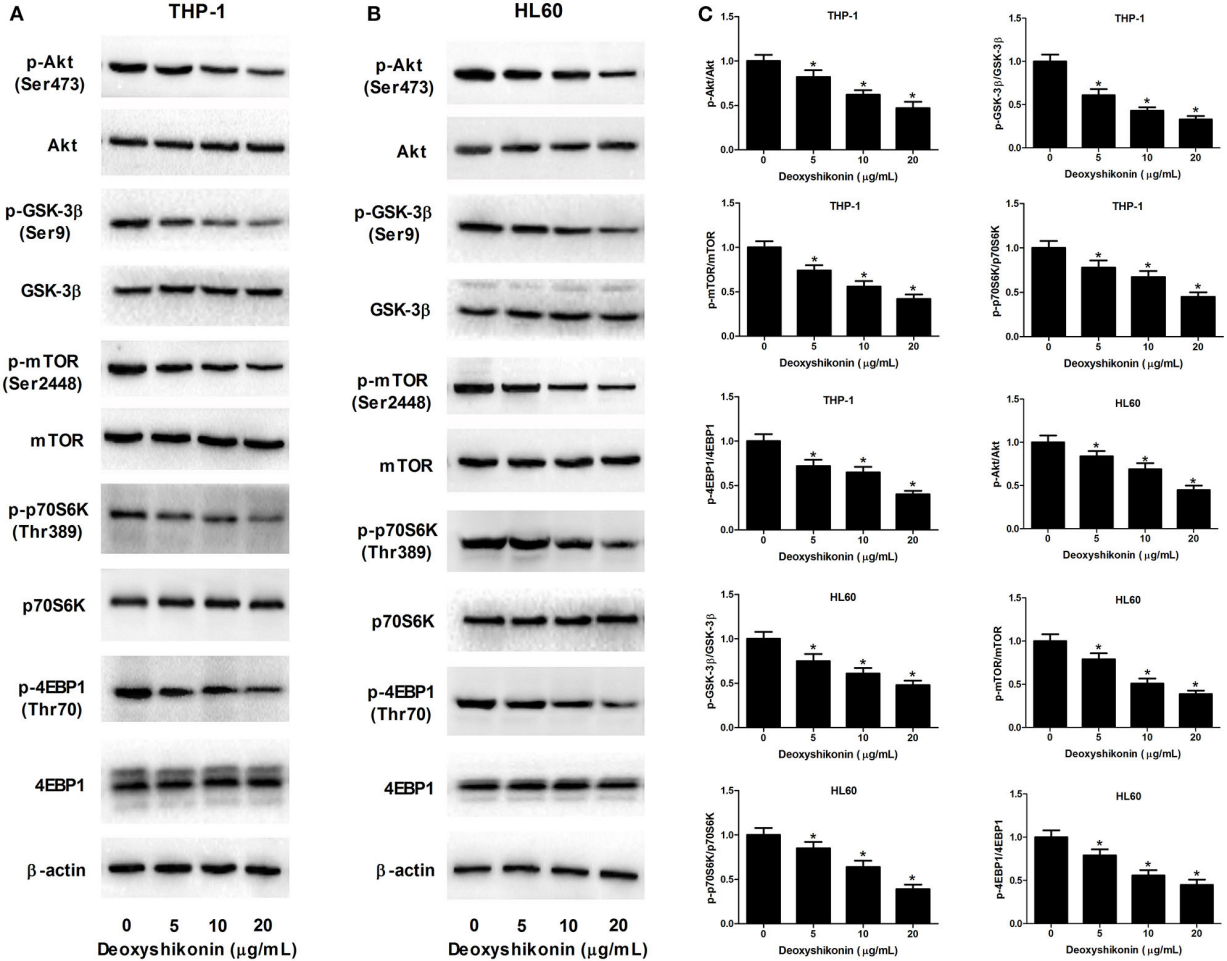
Deoxyshikonin inactivated the Akt/mTOR pathway in AML cells. **(A–C)** THP-1 and HL60 cells were treated with different doses of deoxyshikonin (0, 5, 10, and 20 μg/ml) for 48 h, and the protein levels of p-Akt, Akt, p-GSK-3β, GSK-3β, p-mTOR, mTOR, p-p70S6K, p70S6K, p-4EBP1, and 4EBP1 were measured by western blot analysis. *P < 0.05 compared to the control group.

The aberrant Akt/mTOR signaling has been demonstrated to be associated with the tumorigenesis of miscellaneous cancers including AML (21). We further determined the influence of deoxyshikonin on the Akt/mTOR pathway in AML cells. As demonstrated by western blot analysis, deoxyshikonin treatment restricted the phosphorylation of Akt, GSK-3β, mTOR, p70S6K, and 4EBP1 in a concentration-dependent manner but caused no noticeable change on the total protein levels of Akt, GSK-3β, mTOR, p70S6K, and 4EBP1 in THP-1 and HL60 cells (Figures 6A–C), indicating that deoxyshikonin inactivated the Akt/mTOR pathway in AML cells.

### Activation of the Akt/mTOR Pathway Reversed the Effects of Deoxyshikonin on Viability and Apoptosis of AML Cells

**Figure 7:**
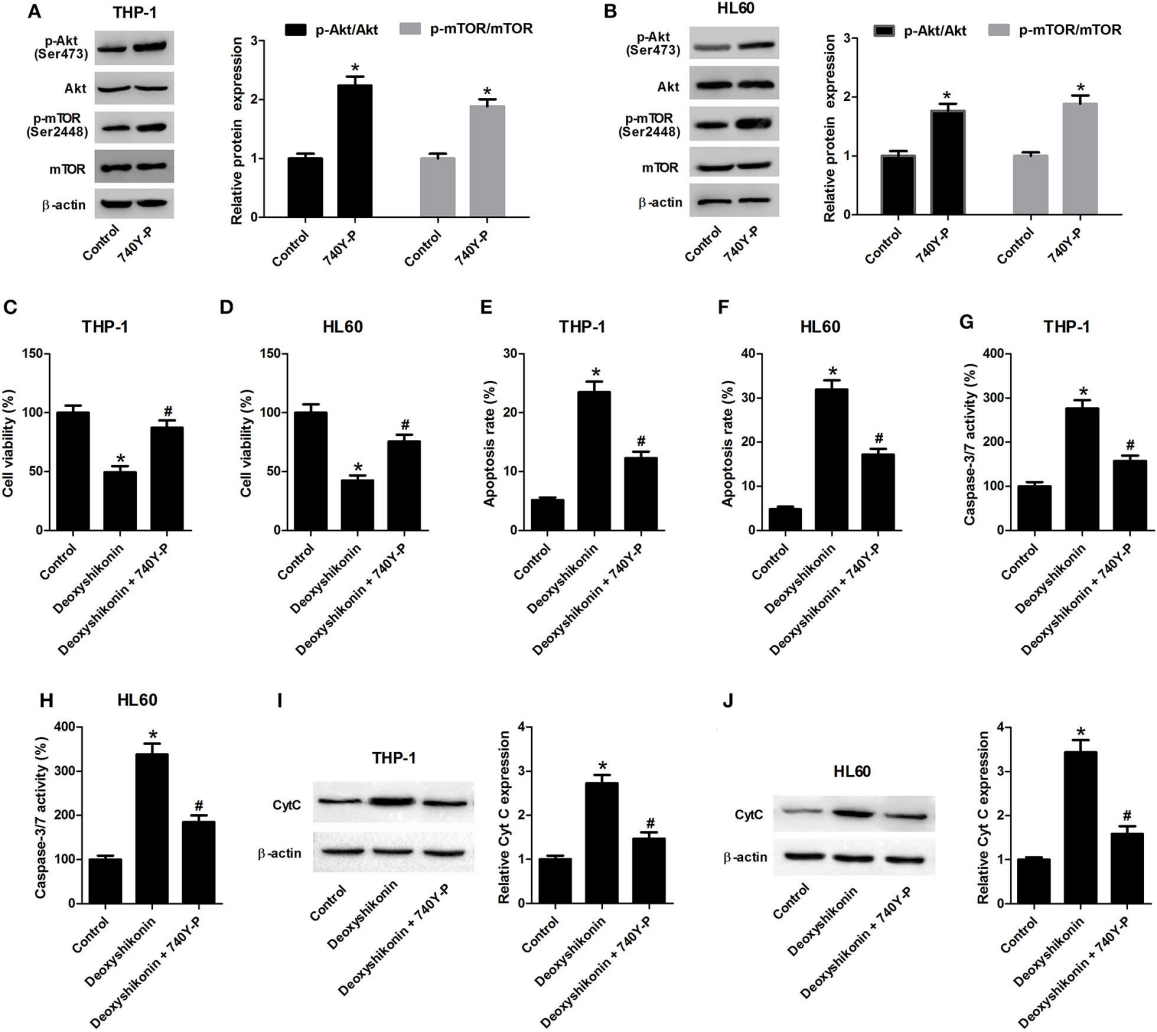
Activation of the Akt/mTOR pathway reversed the effects of deoxyshikonin on the viability and the apoptosis of acute myeloid leukemia cells. **(A,B)** The protein levels of p-Akt, Akt, p-mTOR, and mTOR in the THP-1 and HL60 cells treated with 740Y-P for 48 h were detected by western blot analysis. THP-1 and HL60 cells were treated with 20 μg/ml deoxyshikonin in the presence or the absence of 15 μM 740Y-P for 48 h, followed by assessment of cell viability **(C,D)**, apoptosis **(E,F)**, caspase-3/7 activity **(G,H)**, and Cyt C protein level **(I,J)** by CCK-8 assay, flow cytometry analysis, caspase-3/7 activity assay, and western blot analysis, respectively. *P < 0.05 compared to the control group. ^#^*P* < 0.05 compared to the deoxyshikonin treatment group.

To figure out whether the Akt/mTOR pathway was involved in mediating the anti-tumor effects of deoxyshikonin on AML cells, THP-1 and HL60 cells were treated with 20 μg/ml deoxyshikonin in the presence or the absence of 740Y-P for 48 h. The 740Y-P treatment alone resulted in a notable enhancement of p-Akt and p-mTOR expressions but produced little alternation on Akt and mTOR protein levels in THP-1 (Figure 7A) and HL60 cells (Figure 7B), suggesting the activation of Akt/mTOR signaling by 740Y-P. The CCK-8 assay presented that the deoxyshikonin treatment-induced viability reduction in THP-1 (Figure 7C) and HL60 cells (Figure 7D) was effectively ameliorated following the addition of 740Y-P. The increase of apoptotic rate in deoxyshikonin-treated THP-1 (Figure 7E) and HL60 cells (Figure 7F) was significantly abolished after cotreatment with deoxyshikonin and 740Y-P. Moreover, caspase-3/7 activity was enhanced in THP-1 (Figure 7G) and HL60 cells (Figure 7H) in response to deoxyshikonin, which was attenuated following the addition of 740Y-P. Furthermore, the protein level of Cyt C in the deoxyshikonin + 740Y-P cotreatment group in THP-1 (Figure 7I) and HL60 cells (Figure 7J) was reduced when compared with that of the deoxyshikonin treatment group. Collectively, these results suggested that the activation of the Akt/mTOR pathway reversed the effects of deoxyshikonin on the viability and the apoptosis of AML cells.

### Activation of the Akt/mTOR Pathway Reversed the Effects of Deoxyshikonin on Glycolysis and PKM2 Expression in AML Cells

**Figure 8:**
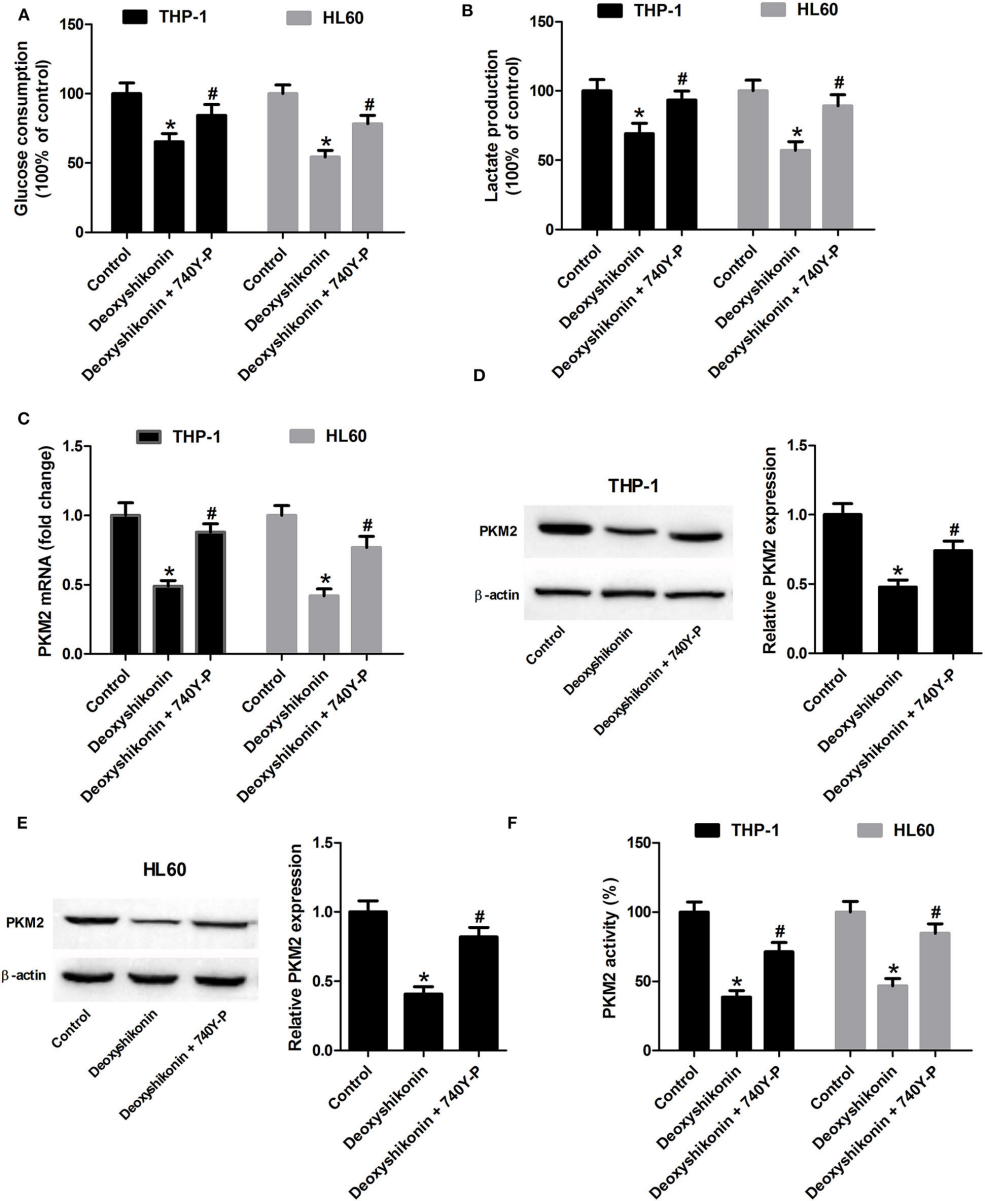
Activation of the Akt/mTOR pathway reversed the effects of deoxyshikonin on glycolysis and PKM2 expression in acute myeloid leukemia cells. THP-1 and HL60 cells were exposed to 20 μg/ml deoxyshikonin or together with 15 μM 740Y-P for 48 h. **(A,B)** Glucose consumption and lactate production in the supernatants of treated THP-1 and HL60 cells were measured. **(C)** The mRNA level of PKM2 in treated THP-1 and HL60 cells was estimated by quantitative real-time polymerase chain reaction. **(D,E)** The protein level of PKM2 in the treated THP-1 and HL60 cells was estimated by western blot. **(F)** PKM2 activity was detected by the lactate dehydrogenase-coupled assay. *P < 0.05 compared to the control group. ^#^*P* < 0.05 compared to the deoxyshikonin treatment group.

**Figure 9:**
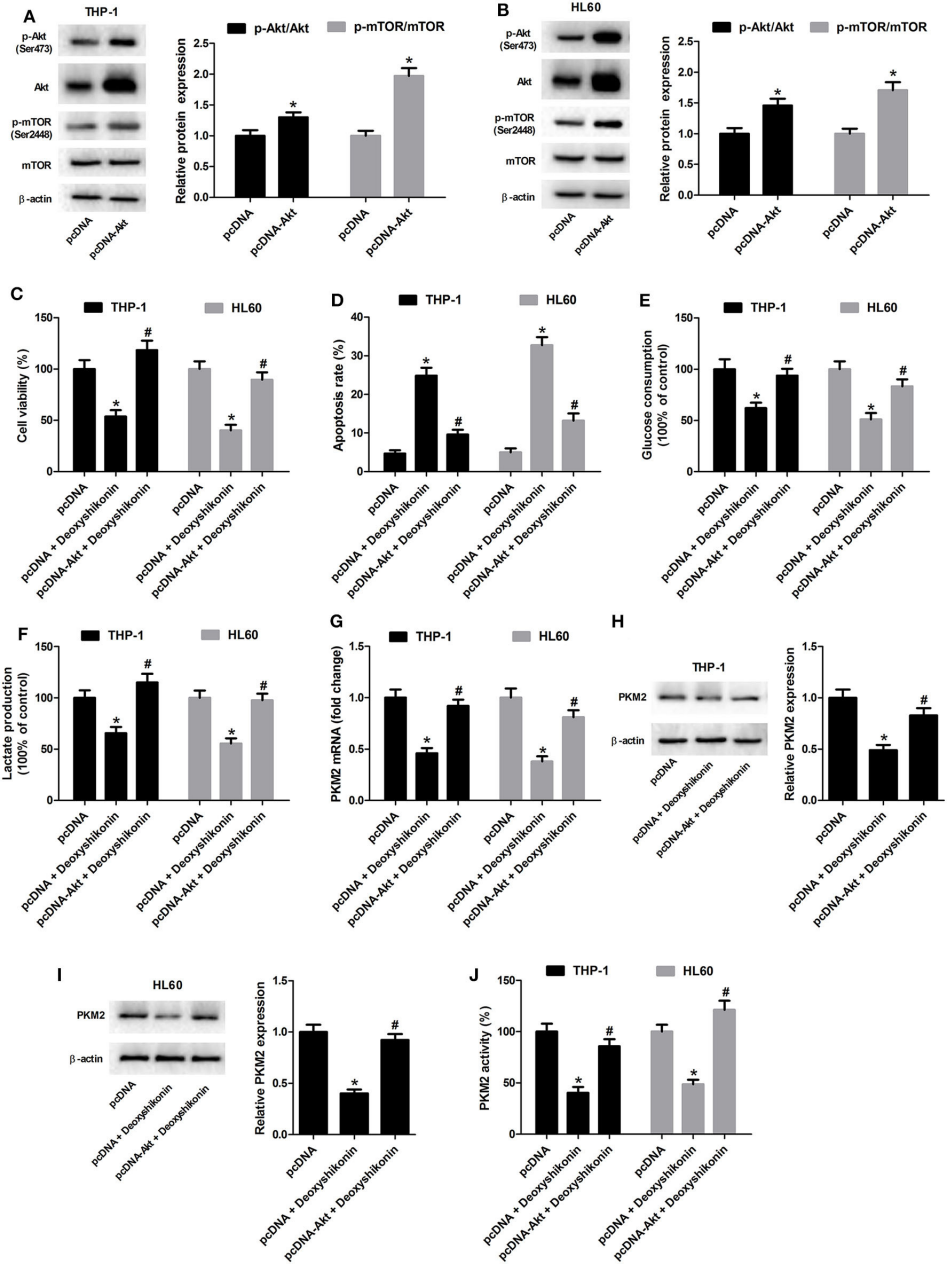
Transfection with pcDNA-Akt reversed the effects of deoxyshikonin on viability, apoptosis, glycolysis, and PKM2 expression in acute myeloid leukemia cells. **(A,B)** The Akt-overexpressing plasmid (pcDNA-Akt) and empty vector (pcDNA control) were transfected into THP-1 and HL60 cells. After 48 h, the protein levels of p-Akt, Akt, p-mTOR, and mTOR were detected by western blot analysis. Transfected THP-1 and HL60 cells were exposed to 20 μg/ml deoxyshikonin for 48 h, followed by assessment of cell viability **(C)**, apoptosis **(D)**, glucose consumption **(E)**, and lactate production **(F)** as well as PKM2 mRNA **(G)**, protein **(H,I)**, and activity **(J)**. *P < 0.05 compared to the pcDNA group. ^#^*P* < 0.05 compared to the pcDNA + deoxyshikonin group.

The 740Y-P treatment overturned the reduction of glucose consumption (Figure 8A) and lactate production (Figure 8B) mediated by deoxyshikonin in THP-1 and HL60 cells. The deoxyshikonin treatment alone significantly suppressed PKM2 mRNA expression in THP-1 and HL60 cells, which was restored by the combined treatment of deoxyshikonin and 740Y-P (Figure 8C). Moreover, 740Y-P resisted the deoxyshikonin-induced decrease of PKM2 protein level (Figures 8D,E) and PKM2 activity (Figure 8F) in THP-1 and HL60 cells. These results suggested that the activation of the Akt/mTOR pathway reversed the effects of deoxyshikonin on glycolysis and PKM2 expression in AML cells. To confirm the abovementioned results, THP-1 and HL60 cells were transfected with Akt-overexpressing plasmid (pcDNA-Akt) to activate the Akt/mTOR pathway. As shown in Figures 9A,B, the ratios of p-Akt/Akt and p-mTOR/mTOR were increased 48 h after transfection in THP-1 and HL60 cells. The CCK-8 assay showed that deoxyshikonin treatment-caused viability reduction in THP-1 and HL60 cells was attenuated after transfection with pcDNA-Akt (Figure 9C). The increase of apoptotic rate in deoxyshikonin-treated THP-1 and HL60 cells was significantly abolished after transfection with pcDNA-Akt (Figure 9D). Transfection with pcDNA-Akt impaired the deoxyshikonin treatment-caused decrease of glucose consumption (Figure 9E) and lactate production (Figure 9F) in THP-1 and HL60 cells. The deoxyshikonin treatment inhibited the expression of PKM2 mRNA (Figure 9G) and protein (Figures 9H,I) and the activity of PKM2 (Figure 9J), whereas these effects were attenuated after transfection with pcDNA-Akt. These results confirmed that the activation of the Akt/mTOR pathway reversed the effects of deoxyshikonin on viability, apoptosis, glycolysis, and PKM2 expression in AML cells.

## Discussion

In spite of significant improvements in therapeutic interventions of AML, the prognosis of patients suffering from AML remains unfavorable and the 5-year survival rate of AML patients is lower than 30%, accompanied by a high mortality rate (22). For the high mortality, there is a great need to identify effective alternative therapeutic agents specifically targeting AML. It is commonly reckoned that natural products have the potential to induce apoptosis in cancer cells including AML and may therefore be essential sources for anticancer drugs because of their extensive biological activities and limited side effects (23, 24). Shikonin and its derivatives, the predominant type of naphthoquinone derivatives extracted from the root of *Lithospermum erythrorhizon* Sieb. et Zucc., have been well-documented to possess a wide range of pharmacological activities, including anti-tumor activity by suppression of cell proliferation. For instance, shikonin potently depressed the viability and the metastasis of triple-negative breast cancer cells by reversing the epithelial-to-mesenchymal transition *via* glycogen synthase kinase 3β-regulated repression of β-catenin signaling (25). Additionally, β-dimethylacrylshikonin suppressed cell viability and induced mitochondria-dependent apoptosis in human lung adenocarcinoma cells *via* the activation of the p38 signaling pathway (26). Acetylshikonin, another shikonin derivative, significantly inhibited the anchorage-independent growth of pancreatic cancer cells by suppressing the nuclear factor-kappa B signaling pathway (27). More importantly, it was previously demonstrated that deoxyshikonin exhibited anti-proliferative and pro-apoptotic activities in colorectal cancer cells through the phosphoinositide 3-kinase (PI3K)/Akt/mTOR pathway (10). Nevertheless, whether deoxyshikonin showed an anti-tumor activity in AML remained far from being addressed. To our knowledge, this is the first time to demonstrate that deoxyshikonin dampened the viability of AML cells in a dose-dependent manner. Meanwhile, we found that exposure to deoxyshikonin led to a concentration-dependent increase of the apoptotic rate, caspase-3/7 activity, and Cyt C protein level in AML cells. The increase of caspase 3/7 activity is an important indicator of apoptosis. These results suggested that deoxyshikonin exerted an anti-tumor activity in AML cells. The effects of deoxyshikonin on the viability and the apoptosis of normal bone marrow stromal HS-5 cells were also evaluated in this study. The results showed that deoxyshikonin, at 20 μg/ml, did not affect HS-5 cell viability and apoptosis (Supplementary Figure 1), suggesting that deoxyshikonin was selectively toxic to cancer cells but not to normal cells.

It has been proven that disruption of aerobic glycolysis restricts cancer carcinogenesis, suggesting that elevated aerobic glycolysis facilitates tumor development and oncogenesis (28). Our study provided evidence that deoxyshikonin inhibited glycolysis in AML cells as demonstrated by decreased glucose consumption and lactate production. Moreover, we found that deoxyshikonin inhibited the expression of PKM2 in AML cells. PKM2, a critical rate-limiting enzyme of aerobic glycolysis, is proposed to play a crucial role in glycolysis process and cancer progression (29). The robust expression of PKM2 has been observed in various human cancers, which facilitates cancer cell proliferation and growth (30). These findings together revealed that deoxyshikonin inhibited glycolysis in AML cells by suppressing PKM2.

It has been demonstrated that the Akt/mTOR signaling network is constitutively activated and associated with the development of several types of cancers including AML (31, 32). Over-activation of the Akt/mTOR signaling is involved in the elevated aerobic glycolysis of cancer cells, thereby contributing to cancer cell survival and growth (33). Thus, the Akt/mTOR pathway may be regarded as a promising therapeutic target for cancer treatment (34). To elucidate the molecular mechanism underlying the anti-tumor effects of deoxyshikonin, we detected the influence of deoxyshikonin on the Akt/mTOR signaling in AML cells. It was shown that deoxyshikonin impeded the activation of the Akt/mTOR signaling in AML cells. In the restoration assay, activation of the Akt/mTOR signaling by 740Y-P or pcDNA-Akt plasmid abolished the anti-tumor effect of deoxyshikonin in AML cells. Taken together, these results suggested that deoxyshikonin dampened the viability and the glycolysis of AML cells by suppressing PKM2 *via* inactivation of the Akt/mTOR signaling. The main defect of this study is that experiments were only performed on two AML cell lines (THP-1 and HL60), and no primary AML cells were tested. The heterogeneity of AML is therefore not taken into account. Future studies should explore the role of deoxyshikonin using primary AML cells.

## Conclusion

To sum up, our study provided the first evidence that deoxyshikonin exerted anti-tumor and anti-glycolytic activities in AML cells by suppressing PKM2 *via* inactivation of the Akt/mTOR signaling. Our study provided novel insights into the anti-tumor and anti-glycolytic activities of deoxyshikonin in AML. Deoxyshikonin may be a promising anticancer candidate agent in AML cells.

## Data Availability Statement

All datasets generated for this study are included in the article/Supplementary Material.

## Author Contributions

HW conducted the experiments and participated in the conception and the design of the study. HZ conducted the experiments and performed the analysis. LC contributed to analyzing the data and drafting the manuscript. All authors contributed to the article and approved the submitted version.

## Conflict of Interest

The authors declare that the research was conducted in the absence of any commercial or financial relationships that could be construed as a potential conflict of interest.
