# Supplementary material for: Deoxyshikonin Inhibits Viability and Glycolysis by Suppressing the Akt/mTOR Pathway in Acute Myeloid Leukemia Cells
Source: Front Oncol. 2020 Aug 7;10:1253. doi: 10.3389/fonc.2020.01253 (PMC7427633; doi:10.3389/fonc.2020.01253)
Supplement: Supplementary file 1 [file Data_Sheet_1.docx]

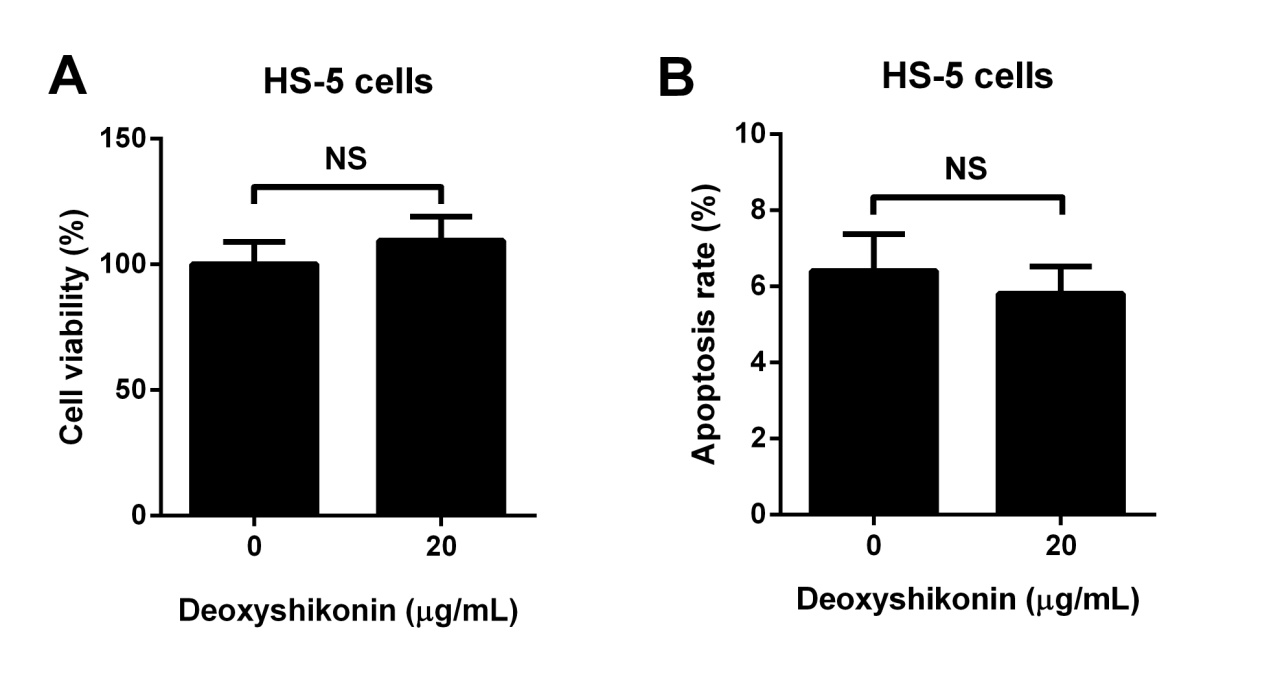


**Supplementary Figure.** Human bone marrow stromal HS-5 cells were treated with 0 or 20 μg/mL deoxyshikonin for 48 h. **(A)** Cell viability was assessed by CCK-8 assay. **(B)** The apoptotic rate was evaluated by Annexin V-FITC apoptosis assay. NS, not significant.
